# Bladder preservation in muscle-invasive bladder cancer: a comprehensive review

**DOI:** 10.1590/S1677-5538.IBJU.2020.99.01

**Published:** 2020-01-10

**Authors:** Judy Hamad, Hannah McCloskey, Matthew I. Milowsky, Trevor Royce, Angela Smith

**Affiliations:** 1 University of North Carolina Chapel Hill School of Medicine Chapel HillNC USA University of North Carolina at Chapel Hill School of Medicine; Chapel Hill, NC, USA;; 2 Department of Urology University of North Carolina Chapel HillNC USA Department of Urology, University of North Carolina at Chapel Hill; Chapel Hill, NC, USA;; 3 Department of Medicine Division of Hematology/Oncology University of North Carolina Chapel HillNC USA Department of Medicine, Division of Hematology/Oncology, University of North Carolina at Chapel Hill; Chapel Hill, NC, USA;; 4 Department of Radiation Oncology University of North Carolina Chapel HillNC USA Department of Radiation Oncology, University of North Carolina at Chapel Hill; Chapel Hill, NC, USA;; 5 Department of Urology Lineberger Comprehensive Cancer Center University of North Carolina Chapel HillNC USA Department of Urology, Lineberger Comprehensive Cancer Center, University of North Carolina at Chapel Hill; Chapel Hill, NC, USA

**Keywords:** Urinary Bladder Neoplasms, Therapeutics, Cystectomy

## Abstract

**Background:**

Standard management of muscle-invasive bladder cancer involves radical cystectomy with pelvic lymph node dissection. However, patients may be ineligible for surgery or may wish to avoid the morbidity of cystectomy due to quality of life concerns. Bladder preservation therapies have emerged as alternatives treatment options that can provide comparable oncologic outcomes while maintaining patients’ quality of life.

**Objective:**

To review bladder preservation therapies, patient selection criteria, and functional and oncologic outcomes for BPT in muscle-invasive bladder cancer.

**Materials and Methods:**

We conducted a comprehensive literature review of bladder preservation therapies in Pubmed and Embase.

**Discussion:**

The ideal patient for BPT has low-volume T2 disease, absence of CIS, absence of hydronephrosis, and a maximal TURBT with regular surveillance. Technological advancements involving cancer staging, TURBT technique, and chemotherapy and radiation therapy regimens have improved BPT outcomes, with oncologic outcomes now comparable to those of radical cystectomy. Advancements in BPT also includes a heightened focus on improving quality of life for patients undergoing bladder preservation. Preservation strategies with most evidence for use include trimodality therapy and partial cystectomy with pelvic lymph node dissection.

**Conclusions:**

This review highlights the breadth of strategies that aim to preserve a patient’s bladder while still optimizing local tumor control and overall survival. Future areas for innovation include the use of predictive biomarkers and implementation of immunotherapy, moving the field towards patient-tailored care.

## INTRODUCTION

Bladder cancer is the ninth most common cancer worldwide, and the second most common genitourinary malignancy (1). At presentation, approximately 70% of bladder cancer cases are non-muscle invasive and 30% are muscle-invasive (2). Whereas most first-line treatments for non-muscle invasive bladder cancer (NMIBC) are bladder-conserving, the typical management of muscle-invasive bladder cancer (MIBC) includes bladder removal with bilateral pelvic lymph node dissection. The addition of neoadjuvant chemotherapy to MIBC treatment results in a 5% absolute improvement in survival at 5 years by reducing micro-metastatic disease at the time of surgery (3), with many regimens including cisplatin-based chemotherapy (e.g. cisplatin) (4, 5).

Although radical cystectomy with neoadjuvant chemotherapy is considered standard treatment for MIBC, the associated morbidity and mortality remain significant concerns (6). The morbidity associated with cystectomy has spurred a growing interest in bladder conserving treatments, such as trimodality therapy (TMT) and partial cystectomy with neoadjuvant chemotherapy. Pooled analyses of prospective cohort studies demonstrated a 5-year overall survival (OS) of 57% and 5-year disease-specific survival (DSS) of 71% following trimodality therapy (7). Similarly, a meta-analysis published in 2015 demonstrated a 5-year OS of 56% with TMT, comparable to the OS seen following RC (8). TMT is also associated with higher quality of life scores, including better social, physical, sexual, and cognitive functioning compared to patients who underwent RC (9). With the growing body of research showing similar efficacy of TMT in properly selected patients, we identify the need to highlight TMT strategies and associated outcomes.

We present a comprehensive review of bladder preservation therapies (BPT) for MIBC, focusing on cT2-T4 N0M0 MIBC unless otherwise noted. Our objective is to review patient selection as well as oncologic and functional outcomes with BPT for MIBC.

## MATERIALS AND METHODS

We conducted a comprehensive search of PubMed and Embase databases on September 12, 2019. Our search included the following United States National Library of Medicine Medical Subject Headings (MeSH) terms: muscle-invasive bladder cancer, bladder preservation, bladder preserving treatments, and organ sparing treatments. We limited our search to articles written in English and excluded conference abstracts. We complemented this search by identifying additional articles referenced in the full-text review stage.

## RESULTS

### Patient Selection Criteria

Patients of two distinct categories have historically undertaken bladder preservation therapy: those who are medically inoperable (unfit for surgery) and those with organ-confined disease who have a strong preference to avoid radical surgery.

The National Comprehensive Cancer Network (NCCN) recommends bladder preservation over RC be reserved for patients whose tumors are small and solitary, lack lymph node metastases, lack carcinoma *in situ* (CIS), are without tumor-related hydronephrosis, and have favorable baseline bladder function (10). There is no absolute size cut-off for tumors amenable to bladder preservation, but it is generally agreed that tumors larger than 5 or 6 cm preclude bladder preservation. Patients with hydronephrosis have lower rates of complete response and 5-year DSS, as well as increased rates of salvage cystectomy, and thus are generally not candidates for bladder preservation (11). Other factors associated with complete response and successful BPT include low-volume T2 lesions, lesions amenable to complete TUR, and normal performance status (Table-1) (12-14). Completeness of TUR, both microscopically and macroscopically, is associated with improved patient outcomes (10), discussed in subsequent sections of this review.

**Table 1:** Bladder Preservation Patient Selection Criteria.

Disease-related Factors	Patient-related Factors
Small, low-volume solitary tumors	Favorable baseline bladder function
T2 disease	Normal or favorable performance status
No CIS	
No hydronephrosis	
No lymph nodes metastases	
Tumor amenable to complete TUR	

With limited selection criteria outlined above, physicians may rely on clinical judgement for patient treatment selection, which can result in utilization disparities between RC and BPT. Analysis of Surveillance, Epidemiology, and End Results (SEER)- Medicare data of patients with cT2 MIBC found that older age at diagnosis and higher comorbidity were associated with decreased utilization of RC (15). Chronological age alone, however, should not preclude a patient from definitive therapy with RC or push a patient towards BPT, though it is often used as a proxy for fitness for surgery given increasing comorbidities with age. To better elucidate the prevalence of comorbidities in an elderly MIBC population, an evaluation of SEER data found that patients above the age of 75 with MIBC were more likely to have prior cancer diagnoses, cardiac disease, chronic anemia, and worse American Society of Anesthesiologists (ASA) Physical Status Classification, as compared to patients less than 75 years of age (16). As the number of comorbidities increases, so does the surgical risk. Thus, using comorbidities and performance status to predict outcomes instead of over-relying on chronological age allows clinicians and patients to make more informed treatment plan decisions.

There has been a surge in research investigating the role of biomarkers to predict a patient’s response to treatment, however, to date, no biomarker has been incorporated into routine clinical decision making outside of clinical trials. Most studies analyzing associations of predictive biomarkers with clinical response in bladder preservation consist of retrospective reviews; thus, conclusions are limited in nature. Biomarkers have been categorized into apoptosis-related, cell proliferation-related, receptor tyrosine kinases, DNA damage response mediated, hypoxia markers, and by molecular sub-types (17). Delving into each of these categories is beyond the scope of this review, but we refer readers to two reviews covering the wide breadth of studied biomarkers in bladder preservation (17, 18). Incorporation of predictive biomarkers is likely a future direction for patient-tailored treatment of bladder cancer.

## SINGLE MODALITY THERAPIES

Experts generally agree that single modality therapies such as radical TUR, chemotherapy, and radiotherapy are less effective alone than in combination for the treatment of MIBC (10). However, some historical series demonstrate efficacy in highly selected patients.

### Maximal Transurethral Resection Alone

TURBT functions as both a diagnostic and therapeutic procedure in the management of bladder cancer. Maximal TURBT, defined as macroscopically complete resection of the bladder tumor when safely possible, is critical to successful treatment in mono- and multi-modality regimens. Guidelines for both NMIBC and MIBC emphasize conducting a maximal TURBT, with resection down to the detrusor muscle when feasible (4, 10, 19). Depending on the size and location of the tumor, however, maximal TUR may not be possible and requires special considerations (20).

Cohort studies of patients receiving TUR alone for T2 (B1 or B2) disease from the 1950s-1970s demonstrated inferior overall survival rates compared to RC, ranging from 31-38% at 5 years (21-23). Later studies documenting the use of TUR monotherapy showed efficacy in cohorts with very specific patient selection, with some studies revealing comparable 5-year survival rates to RC. Solsona and colleagues prospectively followed 133 patients with invasive bladder cancer who were treated with radical TUR and had negative biopsies of the tumor bed; 5- and 10-year cancer-specific survival rates were 80.5% and 74.5%, with bladder preservation rates of 82.7% and 79.6% at 5 and 10 years respectively (24, 25). After 15 years of follow-up in the same cohort, OS was found to be 73.7% at 5 years, 39.8% at 10 years, and 24.8% at 15 years. Although repeat TUR was not systematically performed, only 9 patients (6.7%) had their cancers under-staged, validating the study’s selection criteria (26).

Similarly, Herr followed 99 patients treated with TUR alone for MIBC and 52 who underwent RC; 76% survived by 10 years of follow-up in the TUR group compared to 71% in the RC group (27). He found that mortality from a new invasive tumor during follow-up was 31% for patients restaged as having persistent T1 disease compared with 11% for patients without residual cancer (T0) after restaging TUR (27). More recently, a retrospective review of 327 patients with MIBC treated at MD Anderson demonstrated that only 11% of patients qualified for bladder preservation with TUR. Qualification criteria included patients with no residual tumor on re-resection, normal exam under anesthesia, and absence of upper urinary tract pathology (28). BPT with TUR alone is thus appropriate for only a small proportion of patients with MIBC. Risk of recurrence following TUR monotherapy is shown to be anywhere from 38-56%, emphasizing the need for careful patient selection and regular cystoscopic follow-up (27, 28). Even with highly specific selection criteria, a high rate of recurrence leads to more salvage cystectomies, with a salvage cystectomy rate of 30% seen in the MD Anderson series (28).

If a patient receives TUR alone, NCCN guidelines recommend maximal repeat TUR within 4 weeks to ensure absence of residual disease. If negative, patients should be monitored with repeat cystoscopy and cytology every 3 months; if relapse occurs, the stage at re-resection determines subsequent management (10).

### Radiation Monotherapy

Radiation as a monotherapy for bladder preservation in MIBC was historically undertaken in patients unfit for RC, creating a barrier to comparison with RC outcomes. Fossa and colleagues retrospectively reviewed 263 patients who received RC and 271 patients deemed unfit or unwilling to undergo RC who received high-dose XRT. Five-year OS for the RC group was 48% compared to 22% in the RT group, noting that for each T stage group, survival rate for RC patients was twice that of XRT patients (29). A large retrospective series of 917 patients with transitional cell carcinoma of the bladder (T1-T4) undergoing XRT in the UK showed 5-year OS ranging from 11.6 % (ages >79) to 50.4% (ages <60). Chung et al. stratified OS by stage, finding that OS steadily decreased with increasing stage: 48% T2a, 37% T2b, 21% T3b, 13% T4a, and 8% T4b at 5 years (30). Other series have shown similar rates of OS following RT monotherapy, ranging from 22-40%, and notably all lower than those seen in RC series (30-35). Radiation monotherapy has also been studied as arms of overarching randomized trials. In these trials, radiation monotherapy shows comparable but lower OS compared to RC + radiation or TMT arms, and still remains a reasonable option for patients with comorbidities precluding treatment with modalities such as radical surgery or chemotherapy (36-38).

One study aimed to identify whether targeting radiation to the tumor-bearing region alone, as opposed to conventional whole-bladder radiation, could improve local disease control and thus overall survival while also limiting toxicity. The whole bladder radiation control arm had a 5-year OS rate of 61%, compared to 60% and 51% in the two partial bladder XRT trial arms (p=0.81) (39). Kang et al. similarly found equivalent disease-free and OS in a smaller series comparing whole-bladder versus partial-bladder XRT, but they noted that hypofractionation with partial-bladder RT resulted in one-third reduction in both treatment duration and cost (40). In this study, patients receiving whole-bladder radiation had more acute and late toxicities than the partial-bladder group, although this series did not assess for statistical significance of this difference perhaps given small sample size. Dose escalation from the standard 64-66 Gy has not been found to improve survival and may impose a higher risk of toxicity (41).

Overall survival after XRT differs with location of treatment delivery. One study demonstrated that the composite National Comprehensive Cancer Network (NCCN) guidelines compliance rate of receiving TURBT before XRT, use of concurrent chemotherapy, and total dose of XRT was 48.0% at high-volume RT facilities versus 41.0% for low volume facilities (p<0.0001), with a statistically significant difference in 5-year OS rates (high-volume facilities 24.8%, low volume facilities 20.7%, p=0.001). Among patients whose treatment was compliant with all 3 NCCN parameters, OS remained statistically significantly higher at facilities with high XRT volumes (p=0.029). Accounting for unmeasured socioeconomic confounders is a challenge but these studies raise questions regarding health disparities, as African American patients, rural community-dwelling patients, lower median household incomes, and lower education levels were more likely treated at low-volume RT facilities (42).

### Chemotherapy Monotherapy

There is a limited role for chemotherapy (CT) as the sole agent in the treatment of MIBC. As part of the standard non-preservation therapy for bladder cancer, chemotherapy is typically used in the neoadjuvant setting with RC. When combined with RC and PLND, cisplatin-based chemotherapy results in improved survival (5) with the greatest benefit seen in patients who achieve complete pathological response following NAC (43). There may also be a role for adjuvant chemotherapy following RC (10, 19).

Recently, presence of certain DNA damage response (DDR) gene mutations, associated with sensitivity to cisplatin-based chemotherapy, has spurred investigations into their use as predictive biomarkers of response to chemotherapy in bladder preservation (44). An Alliance for Clinical Trials in Oncology phase II trial at Memorial Sloan Kettering is currently studying whether patients with DDR mutations can forgo RC and PLND and be managed with cisplatin-based chemotherapy alone (45). Chemotherapy in the setting of BPT will be discussed in subsequent sections of this review.

## MULTIMODALITY THERAPIES

Modern multimodal bladder preservation typically involves some variation of maximal TUR with chemoradiation therapy. This is followed by regular cystoscopic evaluation to determine response to therapy, with prompt salvage RC should the patient not respond or have a muscle invasive recurrence.

### TUR + Chemotherapy

The addition of chemotherapy to maximal TUR was an attempt to improve local tumor control and reduce the risk of recurrence that is seen with TUR monotherapy. Early series examining TUR with chemotherapy were small and heterogeneous. One retrospective review examined 50 patients treated with maximal TUR followed by 2 to 6 cycles of adjuvant cisplatin-methotrexate (per the EORTC protocol 30851). Thirty-eight (76%) patients remained tumor-free at a median follow-up of 47 months. Ten patients (20%) relapsed with either Ta, T1 + CIS, or CIS at a median follow-up of 15.6 months, with 60% of these recurrences located at the original tumor site. Overall, the bladder was preserved in 37 (74%) of patients (46).

In a phase II nonrandomized trial, 75 patients with positive biopsies of apparently healthy tumor bed during TURBT subsequently received three cycles of cisplatin-based chemotherapy while the control group of 71 received RC. The bladder-sparing group had 5-and 10-year CSS rates of 64.5% and 59.8%, which were not statistically significantly different from the RC arm (p=0.544). Among 51 patients who initially underwent BPT, 40 (53%) achieved a complete response to therapy, 16 (31.3%) developed recurrence, and 15 (29.3%) developed progression. Of the patients who achieved any clinical response (partial or complete), 56% developed progression or recurrence, resulting in a further 45% requiring RC (47). This series demonstrates that many patients’ undergoing TURBT and chemotherapy will subsequently progress or recur, requiring escalation of therapy with salvage RC.

A systematic review that encompassed 18 publications and 518 patients who received systemic chemotherapy plus TURBT found that OS ranged from 20% to 87.5%, with a median follow-up range of 4 to 120 months. The 5-year OS rate for all patients in this review was 72% (95% CI 64%-82%). However, selection criteria across studies varied, with some patients selected due to lack of fitness for RC and others who elected for BPT (48).

### Trimodal Therapy

Trimodal therapy typically consists of maximal TURBT (as safely as possible) followed by chemoradiotherapy. Acceptance and implementation of TMT by the urologic community has been with caution due to concerns of cancer recurrence and need for salvage RC. This is likely perpetuated by the lack of randomized controlled trials comparing TMT to RC, exemplified by the Selective Bladder Preservation Against Radical Excision (SPARE) trial ending early due to a failure to accrue patients (49). However, numerous retrospective and prospective studies have been conducted that contribute to a growing evidence base (prospective studies summarized in Table-2).

**Table 2:** Prospective trimodal therapy studies.

Series	Study Type & Trial Number	Patient Characteristics	Sample Size	Follow-up	Outcomes	Findings
Hussain et al. 2001 (54)	Prospective, single institution SWOG 9312	Unresectable tumors (34%)	N = 56	Not specified	Complete response N= 26 (49%)	Patients with best survival were ones who were fit for surgery but elected for TMT.
		Medically unfit for surgery (21%)			5-year OS 32% entire cohort, 45% (RC refusal), 31% (medically unfit), 20% (surgically unfit)	Patients who received maximal TUR, as opposed to just biopsies, had better progression free survival
		Refused cystectomy (45%)				
Kaufman et al. 2009 (13)	Prospective, multi- institution RTOG 99-06	Medically operable	N = 80	Median: 49.4 mo	CR = 81%	Addition of paclitaxel to induction and consolidation resulted in greater cancer control, but more grade 3-4 toxicity.
					Of the CR, 18 (28%) had local recurrence 5-year OS: 56%, Acute toxicity: Grade 3 (25%), grade 4 (1%)	
Lagrange et al. 2011 (55)	Prospective, multi-institution GETUG 97-015	Medically operable (n=38); Medically unfit or refused RC (n=15)	N = 53	Median: 8 year	8-yr OS: 36% (overall), 45% (fit for surgery) and 13% (unfit for surgery); Metastasis = 43%; Mean QoL scores slightly improve 6 mo after TMT and maintained for 70% after 12 mo.	QoL found to be high; Patients fit for surgery have better survival than those unfit.
Mitin et al. 2016 (7)	Pooled prospective cohorts, multi-institution NRG Oncology/RTOG 99-06, 02-33	Medically operable	N = 119	Median = 5.9year	CR = 85%, near-complete = 15%; Recurrence rate = 34%; 5-year OS = 72% (complete responders) vs 61% (near-complete responders)	Even those with near-complete but not complete response may be appropriate for BPT.
Michaelson et al. 2017 (56)	Prospective, multi institution RTOG 05-24	Medically inoperable; Unfit for platinum-based CRT	N = 66	Not specified	CR = 72.2% (group 1) vs 67.6% (group 2)	Unfit population had comparable rates of CR and adverse events.

**OS =** overall survival; **DSS =** disease-specific survival; **CR =** complete response; **NAC =** neoadjuvant chemotherapy; **CRT =** chemoradiotherapy; **BPT =** bladder preservation therapy

Regarding the delivery of TMT, chemoradiotherapy can be given as a single course of chemoradiation therapy or as a split-course. A split-course entails induction chemoradiation therapy followed by an interval cystoscopy and biopsy and, if a satisfactory response, consolidative chemoradiation therapy. In cases of persistent or recurrent MIBC, salvage cystectomy (with or without perioperative chemotherapy) is recommended, unless the patient has medical contraindications to radical surgery. Most series define complete response as the absence of: visible tumor, biopsy-proven bladder cancer, and tumor cells on urine cytology.

Radiotherapy administered in TMT has been studied in various approaches. One approach entails hypofractionation, in which a total dose of radiation is divided into larger fractions when given over a shorter time period. Hypofractionation protocols include variations in the total dose (Gy), the number of fractions, and the number of days radiation is administered. A prospective phase II trial evaluated the use of concurrent weekly gemcitabine with daily radiation for a total of 52.5 Gy in 20 fractions (50). Another approach involves intensity modulated radiation therapy (IMRT), in which the radiation delivered is manipulated to conform to the shape of a tumor in order to reduce toxicity and maximize the therapeutic ratio (51). A retrospective analysis of 2527 patients in the National Cancer Data Base who received XRT or CRT found that those who received IMRT had improved OS on multivariate analysis compared to those who did not (HR 0.85, 95% CI 0.75-0.97, p=0.02) (51).

With respect to overall performance, TMT has produced comparable oncologic outcomes to RC in appropriately selected patients (8, 52, 53). A systematic review conducted in 2014 found 5-year CSS and OS with TMT to range from 50-82% and 26-74% respectively, with salvage RC rates of 25-30% (53). The similarity in outcomes is likely enabled by promptness of salvage cystectomy when TMT fails. An important distinction when evaluating TMT series is whether patients included in the studies were cystectomy candidates (e.g. medically operable) or not.

A phase II Southwest Oncology Group trial (SWOG 9312) included patients who had surgically unresectable tumors (34%), were medically or surgically unfit (21%), or refused cystectomy (45%). Of note, only some patients received maximal TURBT (39%), with most having only a tumor biopsy conducted during resection (61%). Patients received 4 days of 5-fluorouracil with cisplatin on day 1; this was repeated every 28 days for two total courses during RT as well as two additional courses 4 to 8 weeks after RT was completed. Patients received RT 5 days a week for a total daily dose of 150-200Gy; 50Gy was delivered to the bladder, the prostate and prostatic urethra in men, the urethra in women, and external and internal iliac nodes. An additional 10Gy was delivered to the entire bladder and gross tumor volume. Of the 53 total patients, 26 (49%) achieved a complete response. Five-year OS was 32% for the entire cohort, and when stratified by reason for undergoing TMT, was 45%, 31%, and 20% for RC refusal, medically unfit, and surgically unfit patients, respectively. Patients with maximal TURBT had 38% 5-year progression-free survival, compared to only 14% for patients who only received biopsies during resection (54). This demonstrates the utility of maximal TURBT, when safely possible, and the importance of maximal TURBT in considering the prognosis of a patient undergoing TMT.

A multicenter prospective study (GETUG 97-015) stratified 53 patients into two groups: surgical candidates (n=38) versus those who had a medical contraindication for surgery or who refused surgery (n=15). Maximal TURBT was attempted for all patients but deemed complete for only 33 (66%) patients. All patients received RT with a 45Gy dose in 25 fractions over a period of 4weeks. Potentially operable patients underwent TUR after XRT ended, with salvage cystectomy if persistent tumor was present. Patients unfit for surgery, as well as patients without residual tumor on TUR, received an XRT boost of 18Gy to the bladder with concomitant cisplatin and 5-fluorouracil during weeks 1, 4, and 7 of XRT. Patients who were initially identified as surgical candidates had statistically significantly improved 8-year overall survival of 45% (95% CI 28%-61%), compared to 13% (95% CI 2%-35%) in those who were unfit or refused radical surgery (p=0.001) (55).

A prospective cohort (RTOG 0524) included 66 patients with T2-T4 NXM0 disease who were deemed medically inoperable. Treatment regimens were divided as followed: group (1) immunohistochemical (IHC) 2+ or 3+ expression of Her2/neu received RT + paclitaxel + trastuzumab (n=20; 30%) and group (2) IHC negative or 1+ Her2/neu expression patients received RT+ paclitaxel (n=46; 70%). XRT was done with daily 1.8Gy fractions, 5 days a week, for a total dose of 64.8Gy. All patients received “thorough” TURBT. Complete response rates at 1 year were 72.2% for group 1 and 67.6% for group 2. Treatment-related toxicities were seen in 35% of group 1 and 30% of group 2 patients, with notably low rates of cardiac and hematologic toxicities. Of note, patients were accrued over a seven year period in this study, highlighting the difficulty that such series have faced in accruing patients with medically inoperable bladder cancer (56).

Numerous retrospective and prospective series have studied TMT in medically operable patients. We include select reviews with 100 or more patients. From 1985 to 2001, the RTOG accrued 415 patients considered candidates for cystectomy, across 6 protocols (RTOG 88-12, 88-02, 89-03, 95-06, 97-06, 99-06). The protocols combined TURBT and RT, with varying combinations of cisplatin, methotrexate, vinblastine, paclitaxel, and gemcitabine given in split-course fashion. Complete response rates ranged from 59-75% with 5-year OS 49-52% (57). An updated and pooled analysis of the RTOG 99-06 and 22-03 protocols published in 2016 found that among 119 patients, 85% achieved a complete response (T0 on restaging TURBT) and 15% achieved a near-complete response (Ta or Tis) after induction CRT. These patients then received consolidation XRT of at least 60Gy; incomplete responders proceeded to salvage RC. 36% of the complete responders versus 28% of the near-complete responders had a bladder cancer recurrence at a median follow-up period of 5.9 years, which was not a statistically significant difference (p=0.52). Among all 41 recurrences, 14 (34%) were invasive and resulted in salvage RC; there was no difference in invasive recurrence rates between the complete and near-complete responder groups. 5-year OS was 72% (95% CI 63-81%) for complete responders and 61% (95% CI 39%-84%) for near-complete responders (p=0.12).^7^ This analysis suggests that patients who have a near-complete response with Ta or Tis on restaging TUR are still appropriate candidates for selective bladder preservation, with no difference in recurrence or OS rates.

An unblinded, randomized controlled trial recruited 458 patients across 45 centers in the UK. Patients were randomized in 1:1 fashion to receive radiotherapy with or without synchronous chemotherapy (fluorouracil and mitomycin C) and either whole-bladder radiation or “modified-volume” radiation to the unaffected bladder. The study utilized two radiotherapy fractionation schedules, either 55Gy in 20 fractions over 4 weeks or 64Gy in 32 fractions over 6.5 weeks. The study did not note whether TURBT was maximal. Two-year local disease-free survival was 67% (95% CI 59-74) in the chemoradiotherapy arm compared to 54% (95% CI 46-62) in the radiotherapy arm. The chemoradiotherapy arm had a 2-year relapse rate of 18% compared to 32% in the radiotherapy group (HR 0.57, 95% CI 0.37-0.90, p=0.01). This randomized study demonstrated the added benefit of chemotherapy and that this benefit was not significantly different between the two radiation schedules administered (38).

The Massachusetts General Hospital group retrospectively analyzed 475 patients who underwent TMT by choice and received maximal TURBT with split-course CRT. Only two of the included protocols incorporated NAC (MGH 180 and MGH 880/RTOG 8903 Arm 1), consisting of two methotrexate, vinblastine, and cisplatin (MVC) cycles. Most protocols used a 64-65 Gy RT dose. Seventy-five percent of patients achieved complete response to induction CRT. When stratified by completeness of TURBT, 84% of patients who had complete TURBT achieved CR versus 58% with visibly incomplete TURBTs. Five-, 10-, and 15-year OS rates were 57%, 39%, and 25% respectively. Most patients had T2 disease (66%) and achieved a statistically significantly higher CR rate (83%) as compared to patients with T3-4 disease (63%) (p<0.001). This analysis also stratified outcomes by treatment decades (1986-1995, 1996-2005, and 2005-2013). The CR rate improved from 66% in the 1986-1995 cohorts to 88% in the patients treated from 2005-2013. Furthermore, 5-year OS increased from 53% to 75% from the earliest to latest treatment decades, attributed to improvements in cancer staging, TURBT technique, and chemotherapy and radiation therapy regimens. Salvage cystectomy rates decreased from 42% to 16% across this same time (58).

**Figure 1:**
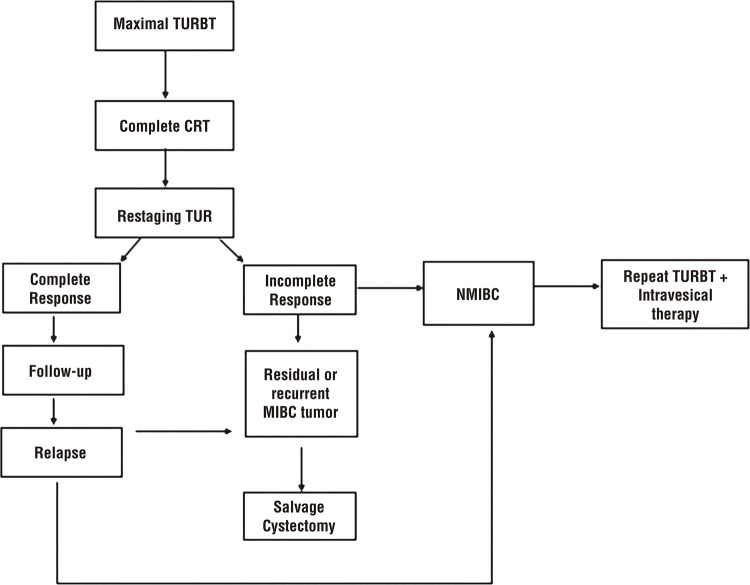
Trimodal therapy treatment algorithm used at our institution, demonstrating an approach for continuous CRT and follow-up.

These series demonstrate comparable oncologic outcomes to RC. One major difficulty in comparing outcomes involves heterogeneous CRT regimens as well as varying surveillance protocols. At our institution, we have historically used a single-course of chemoradiation to 60-64Gy with either 5-fluouracil plus mitomycin or twice-weekly gemcitabine, based on available evidence (Figure-1) (38, 50, 59, 60). Our surveillance protocol consists of cystoscopy every three months for the first two years, every 6 months for years 3-5, and then annually until year 10.

### Partial Cystectomy

The rationale for partial cystectomy (PC) rests in the ability to thoroughly resect bladder masses while still preserving a patient’s bladder and sexual function. With this procedure, as compared to maximal TUR, a surgeon has the ability to more completely assess surgical margins as well as perform PLND if indicated. Factors associated with poor oncologic outcomes following PC include presence of positive pelvic lymph nodes, lymphovascular invasion, need for ureteral reimplantation, and urothelial histology (61-63).

Early PC series produced questionable outcomes and tempered eagerness to pursue it as a bladder-sparing approach. For series conducted in the 1970s-1980s, 5-year OS rates ranged from 25%-48% (64-66), while rates of local recurrence remained high around 54-78% (67-69). Latter PC series with more defined patient selection criteria revealed promising outcomes comparable to RC, although most series had small sample sizes. Across these series, 5-year OS ranged from 53.7% to 70%, and locally advanced tumor recurrence rates ranged from 18.5% to 38% (Table-3) (62, 63, 70, 71).

**Table 3:** Partial cystectomy series identified in this review.

Series	Study Type	Eligibility / Patient Characteristics	Sample Size	Follow-up	Outcomes	Findings
Holzbeierlein et al. 2004 (62)	Retrospective, single institution	Assessed cT/pT stage, age, size of primary tumor, concomitant CIS, margin status, multifocality, and systemic or NAC.	N = 58	Mean: 33 mo	5-year OS = 69%; Superficial recurrence = 12%; Advanced recurrence = 38%	Concomitant CIS and lymph node involvement predictors of advanced recurrence.
Ebbing et al. 2018 (63)	Retrospective, single institution	cT2 only; Medically unfit for RC or elected BPT	N = 27	Median: 36.5 mo	5-year OS = 53.7%; Local recurrence = 18.5%; Salvage cystectomy = 18.5%	Less stringent selection criteria showed worse but comparable outcomes.
Smaldone et al. 2008 (70)	Retrospective, single institution	solitary primary T2 (68%) or T1HG (32%)	N = 25	Mean: 45.3 mo	5-yr recurrence-free, DSS, and OS = 64%, 84%, and 74%, respectively. Locally advanced recurrence = 20%	Only tumor size at time of PC significantly associated with tumor recurrence.
Kassouf et al. 2006 (71)	Retrospective, single institution	Solitary tumor, no CIS, amenable to 2cm margin resection without need for ureteral reimplantation, normally functioning bladder	N = 37	Mean: 72.6 mo	5-year overall, disease specific and recurrence-free survival rates = 67%, 87% and 39%, respectively; Advanced recurrence = 24%	Highly selected cohort
Bazzi et al. 2014 (72)	Retrospective, single institution	Solitary tumor <5cm	N = 36	Median: 16.8 mo	CR to NAC = 58%; Downstaging after NAC = 74%; 5-year recurrence-free, advanced recurrence-free, and overall survival = 28%, 51%, and 63%; Advanced recurrence = 42%	NAC prior to PC associated with acceptable oncologic outcomes.
Kijima et al. 2019 (74)	Prospective, single institution	Tumor circumscription <25% bladder surface, absence of bladder neck involvement, absence of CIS, demonstrated CR to induction CRT	N = 107	Median: 48 mo	5-year DSS and OS = 93% and 91%; PC-related complications = 32%	Tetramodal therapy associated with excellent oncologic and QOL outcomes.

**OS =** overall survival; **DSS =** disease-specific survival; **NAC =** neoadjuvant chemotherapy; **CRT =** chemoradiotherapy; **PC =** partial cystectomy; **BPT =** bladder preservation therapy; **QOL =** quality of life.

The Memorial Sloan-Kettering group published the largest available series consisting of 58 patients who received PC from 1995-2001, finding that 5-year OS was 69% at a mean follow-up of 33 months. Superficial recurrence occurred in 7 patients (12%) and was associated with CIS and tumor multifocality on univariate analysis. Advanced recurrence occurred in 15 patients (38%) and was associated with positive surgical margins and lymph node involvement on univariate analysis. Only concomitant CIS (odds ratio 7.05, p=0.004) and lymph node involvement (odds ratio 4.38, p=0.031) predicted advanced recurrence (62).

Because PC incurs a risk of leaving behind foci of cancer in the bladder with subsequent high rates of local recurrence, some series have attempted to combine PC with other modalities. A retrospective review included 36 patients who received neoadjuvant chemotherapy followed by partial cystectomy. Five-year OS was 63%, with 19 (53%) having recurrence at last follow-up and 22 (61%) maintaining an intact bladder. Positive lymph nodes on imaging and positive surgical margins at PC were associated with lower recurrence-free and OS on univariable analysis. Of note, this series only included patients with solitary tumors of less than 5 cm (72).

TUR with chemoradiotherapy followed by consolidative PC—sometimes referred to as tetramodal therapy—is gaining popularity for its multimodal approach in a highly selected patient population. In one bladder-sparing protocol consisted of debulking TUR and low dose chemoradiotherapy followed by partial cystectomy with PLND in 46 highly selected patients, five-year CSS and recurrence-free survivals were 100%, although histologic examination of PC specimens revealed residual MIBC in 3 (7%) specimens. Median total International Prostate Symptom Scores (IPSS) from 33 of the PC patients was 5 (IQR 2-8.5), at a median follow-up time after PC of 23 months (IQR 10-53); this was reported to be noninferior to a similar population of community-based men in their 70s (73). More recently, a single-institution prospective cohort of 154 patients with T2-T3N0M0 disease initially provided patients with maximal TURBT followed by induction chemoradiotherapy (40Gy in 20 fractions with concurrent cisplatin). Patients who showed complete remission were then offered PC with PLND. Of the 107 who both qualified for and underwent PC, 19 (18%) experienced bladder cancer recurrence with 4 (4%) having MIBC recurrence. Five-year MIBC recurrence-free survival, CSS, and OS were 97%, 93%, and 91%, respectively. QOL survey of the patients who received PC revealed an average IPSS of 2, with the majority of patients mostly satisfied (74).

An analysis of Surveillance, Epidemiology, and End Results (SEER) data in 2009 consisted of 1573 patients treated with PC and 5670 patients treated with RC, covering a wide range of T(1-4)N(1-2)M0 bladder cancer. In this cohort, 5-year OS and CSS estimates for PC patients were 57.2% and 76.4% respectively. Five-year OS and CSS estimates for RC patients were 50.2% and 65.8% respectively. After matching for age, race, tumor stage, tumor grade, nodal status, and year of surgery, 5-year OS and CSS rates were 56.0% and 73.5% for PC, and 54.6% and 69.2% for RC. These data show that PC and RC have comparable oncologic outcomes at five years (75). In the Ontario Cancer Registry, 3320 patients received PC and 3139 patients received RC from 1994 to 2008. Factors associated with receiving PC included older age, having moderate comorbidities, and receiving surgery outside of a comprehensive cancer center (76). After adjusting for age, comorbidity score, stage, and presence of lymphovascular invasion, PC showed comparable and statistically nonsignificant differences compared to RC, with 5-year OS (HR 0.95, 95% CI 0.79-1.14) and CSS (HR 0.87, 95% CI 0.7-1.09).

### Adverse Effects and Quality of Life Considerations

Bladder preservation strategies come with their own set of complications and considerations. Most TMT series demonstrate acceptable toxicities, although some of the toxicities that do occur can become lifelong concerns of patients. On systematic review, rates of grade 3-4 acute toxicities ranged from 10% to 36%, although this was higher in studies that incorporated perioperative neoadjuvant or adjuvant chemotherapy. The majority of toxicities were gastrointestinal/genitourinary-related. Late grades 1 and 2 toxicities fell across a range of 10%-25% for genitourinary and 5%-6% for gastrointestinal among reporting studies. However, these ranges may underestimate the true prevalence of toxicities as these studies mostly involved physician-reported, as opposed to patient-reported, assessments (53).

Quality of life concerns have been one of the driving forces for studying bladder preservation strategies, and TMT studies have demonstrated comparable—if not superior—quality of life measures. This is largely driven by patients’ ability to retain their native bladders, with bladder retention possible in 70% of patients treated with TMT in a large cohort detailed earlier (58). The GETUG cohort discussed above noted that 35% of their patients reported satisfactory bladder function at baseline before TMT, and at 6, 18, and 36 months after treatment, 43%, 57%, and 29% of patients reported satisfactory function respectively. The LENT-SOMA scale was also used, graded from 0 (no toxicity) to 4 (treatment-refractory toxicity). They reported no grade 4 toxicities; 90% of patients remained free from grade 1 side effects related to dysuria, hematuria, and incontinence after 6 months; 5% of patients had grade 2 urinary frequency (2-3 hour interval between urination) and 10% had grade 3 urinary frequency (1-2 hour interval between urination) (55). In one phase 3 trial, differences in grade 3 or 4 toxicities trended toward significance among patients who underwent CRT versus RT-only as part of their multimodal therapy, with 36% in the CRT group compared to 27.5% in the RT group (p=0.07) (38).

A cross-sectional, bi-institutional study surveyed 226 patients with T2-T4 MIBC who were treated with TMT from 1990-2011, with a response elicited from 173 (77%). Multivariable analysis showed that TMT patients had a 9.7 point higher QOL (out of 100 points) compared to RC patients (p=0.001). Furthermore, TMT patients had significantly higher physical, emotional, social, and cognitive functioning (6.6-9.9 points; p<0.04), superior bowel function (+4.5 points; p=0.02), and fewer bowel symptoms (-2.7-7.01 points; p<0.05). This study was limited by nature of the heterogeneous follow-up times and not accounting for baseline QOL scores (9).

Finally, a comparative effectiveness analysis developed a Markov model to compare TMT with RC. This study found an increase in 0.59 quality-adjusted life years (QALYs) when undergoing TMT as compared to RC. Sensitivity analysis attributed this gain in QALY to significantly better quality of life associated with TMT in the presence of similar survival rates in the different treatment strategies (77). This study puts QOL into the measure of QALYs, which is important when considering the use of TMT versus RC at a population-level perspective.

## CONCLUSIONS

Interest in bladder preservation techniques has grown significantly over recent years as technological advancements improve BPT outcomes and the focus on improving quality of life heightens. This review highlighted the breadth of strategies that aim to preserve a patient’s bladder while still optimizing local tumor control and overall survival. Trimodal therapy has the most evidence for its use, with newer series demonstrating promising oncologic outcomes including cancer-specific and overall survival. This holds especially true in cohorts of highly selected patients, with the ideal patient for BPT having low-volume T2 disease, absence of CIS, absence of hydronephrosis, and a maximal TURBT with regular surveillance. Although we are unlikely to see randomized controlled trials comparing TMT to RC, as evidenced by the SPARE trial failing to adequately accrue patients, there are many avenues to refine, advance and demonstrate the efficacy of TMT. Future directions and exciting areas of advancement include the use of tetramodal therapy, the use of predictive biomarkers such as DDR gene mutations, and the promise of immunotherapy (78) with subsequent bladder preservation. Regarding immunotherapy, several immune checkpoint inhibitors have gained approval in the last few years, with several trials underway studying combinations of immune-checkpoint inhibitors, their use with chemotherapy, and the potential use in a neoadjuvant setting (79).
